# Investigating Structural Relationships between Professional Identity, Learning Engagement, Academic Self-Efficacy, and University Support: Evidence from Tourism Students in China

**DOI:** 10.3390/bs14010026

**Published:** 2023-12-28

**Authors:** Qian Chen, Qingchuo Zhang, Fenglong Yu, Bing Hou

**Affiliations:** School of Tourism and Cuisine, Yangzhou University, Yangzhou 225127, China; qian.chen@yzu.edu.cn (Q.C.); mx120231296@stu.yzu.edu.cn (Q.Z.); 006541@yzu.edu.cn (F.Y.)

**Keywords:** professional identity, academic self-efficacy, learning engagement, university support

## Abstract

In order to foster students’ development and enhance the training quality within tourism programs at universities, this study aims to investigate the relationships among tourism students’ professional identity, academic self-efficacy, learning engagement, and university support. Professional identity refers to learners’ recognition and understanding of their study programs and is viewed as a dynamic, progressive process consisting of professional cognition, professional emotion, and professional appraisal. Data were collected from 333 tourism students studying at Chinese universities. They were analyzed through SPSS and SmartPLS. The results revealed that there is no significant correlation between students’ professional cognition and learning engagement. However, students’ professional emotions and professional appraisals positively influence learning engagement. Moreover, all three dimensions of professional identity exhibit positive effects on students’ academic self-efficacy. Additionally, students’ academic self-efficacy demonstrates a positive impact on learning engagement, and university support is associated with increased learning engagement and academic self-efficacy. This study contributes to a comprehensive understanding of the learning experience of tourism students and aims to facilitate the advancement of tourism education through cultivating students’ professional identity towards tourism and developing students’ career commitment in the tourism industry. Theoretical and practical implications are discussed.

## 1. Introduction

Higher education represents a pivotal period during which students engage in professional studies and formulate career aspirations. As of 2023, China boasts the world’s largest higher education system [1]. The gross enrollment rate in higher education stood at 59.6% in 2022, encompassing a substantial 46.55 million students [2]. Concurrently, in tandem with China’s burgeoning tourism industry, tourism education is experiencing rapid growth. Notably, the number of Chinese universities offering tourism programs ranks among the highest globally [3]. Despite the significant demand for professional talents generated by the booming tourism industry [4], this demand has not translated into a robust attraction for students in higher education. Students enrolled in tourism programs exhibit a relatively low professional identity, contributing to a persistently high rate of program changes. Moreover, those who persist in tourism programs grapple with issues such as low learning engagement, a lack of academic self-efficacy, and weakened endogenous learning motivation.

Tourism management, classified as a secondary discipline under business administration, has faced dual constraints of operational systems and insufficient support since its inception in China [5]. The escalating competition among various programs within China’s higher education landscape has led to a survival crisis for tourism programs. Presently, many Chinese colleges and universities with tourism offerings contend with the suspension or reduction of enrollment [6], resulting in an inadequate supply of qualified tourism professionals. This scarcity of skilled individuals, despite the heightened demand within the tourism industry, poses a significant threat to the sustainable development of the sector [7].

Professional identity serves as a pivotal indicator reflecting students’ internal psychological state [8]. According to Yu et al. [3], professional identity is not only a significant measure of students’ individual values but also a crucial indicator of their career development goals. Learning engagement is regarded as an evaluative metric for students’ experiential growth and a predictor of higher education quality. While considerable research has been devoted to the learning engagement of university students [9], there is a noticeable dearth of attention given to the engagement levels of students in tourism programs [3]. Additionally, academic self-efficacy stands out as the primary determinant shaping individuals’ goal-setting and actions in the realm of learning. It not only has the potential to motivate students in their learning activities but also triggers positive behaviors. Despite some studies confirming the influence of professional identity on learning engagement [10,11,12], few have explored the intricate relationships among professional identity, academic self-efficacy, and learning engagement. Moreover, scant research has been conducted to scrutinize the specific dimensions of professional identity, leaving uncharted territory regarding how students’ professional identity impacts their academic self-efficacy and learning engagement.

Furthermore, the university environment, deemed significant for students’ learning and living experiences, not only reinforces motivation but also exerts an influence on their learning behavior [13]. Recognized as a catalyst for enhancing learning commitment [14] and shaping students’ mentality and behavior [15], university support remains a central focus in current research, albeit predominantly centered on identifying the aspects in which universities support students [16,17]. Consequently, the extent and manner in which university support influences students’ academic self-efficacy and learning engagement remain uncertain. Given the multitude of factors influencing students’ learning engagement, there is an urgent need to comprehensively explore the synergistic effects of both endogenous and exogenous factors. Thus, to foster students’ development and enhance the training quality within tourism programs, this study endeavors to examine the relationships among professional identity, academic self-efficacy, learning engagement, and university support.

To achieve the research aims and objectives, the remaining paper is presented as follows: First, research hypotheses and a theoretical framework are proposed based on the literature review of professional identity, academic self-efficacy, learning engagement, and university support. Second, the methodology is presented. Third, an analysis of the results is presented, followed by discussions and implications. The research limitations and future research directions are also introduced.

## 2. Theoretical Background and Hypotheses

### 2.1. Professional Identity

The word “identity” first originated from Freud, and it has developed into a very rich connotation, involving multiple dimensions such as society, politics, self, and others [18]. Professional identity is not only a subordinate branch of identity but also a part of social identity and collective identity [19]. Most research focuses on the self and the social affirmation gained in the professional field [20]. With the gradual clarification of the subject, the concept of professional identity is defined as learners’ understanding of their study programs and their positive attitude toward acceptance, thereby achieving a balance between inner and outer reality. This psychological process is often closely related to the changes and development of the programs [21]. In some studies, professional identity refers to learners’ acceptance and recognition of their programs from the heart and is transformed into external behavioral motivation. It is a process of gradual transfer of emotion, attitude, and even cognition, which is divided into three dimensions: professional cognition, professional emotion, and professional appraisal [3,22]. Learning and understanding knowledge are the basis for students to form an identity with their programs [23]. Cognition forms external behavior and internal emotion and is reflected in students’ professional practice [24]. 

### 2.2. Self-Efficacy

Academic self-efficacy represents a specialized application of self-efficacy within the realm of pedagogy, with its conceptual origins rooted in Bandura’s work. Scholars have arrived at a consensus regarding its definition. Specifically, self-efficacy is construed as individuals’ beliefs in their capacity to successfully attain goals and navigate the environmental factors influencing their lives [25]. It stands as a pivotal proximal determinant of behavior [25,26,27]. Academic self-efficacy, an extension of self-efficacy, constitutes a subjective assessment of one’s capabilities, encompassing self-efficacy in learning ability and self-efficacy in learning behavior [28]. Numerous studies indicate that individuals possessing high academic self-efficacy tend to invest more considerable effort in completing academic tasks. Conversely, those with low academic self-efficacy tend to shy away from academic challenges perceived as surpassing their capabilities [29,30]. According to Bandura’s theory of self-efficacy perception, students’ prognostications regarding their aptitude for learning activities significantly influence their motivation, behavior, and academic performance [31]. Serving as a crucial intermediary in students’ conduct [32], academic self-efficacy plays a vital role across all facets of the learning process [33].

Professional identity and academic self-efficacy serve as indicators reflecting students’ cognitive and emotional orientations toward their programs. Tushnova et al. [34] discovered variations in students’ professional identity and academic self-efficacy levels across different study stages. Existing research consistently reveals a direct and significant impact of professional identity on academic self-efficacy, establishing a positive correlation between the two [35]. Chen et al. [8] posit that heightened professional identity corresponds to increased academic efficacy. Strengthened professional identity fosters an inclination among students to showcase their professional worth, leading to positive evaluations and, consequently, an augmentation of academic self-efficacy [12]. In a study by Zhou [36], the mediating role of academic self-efficacy between professional identity and learning engagement among nursing students was investigated, affirming the positive influence of professional identity on academic self-efficacy. This perspective has found support among students enrolled in special education programs as well. Building upon the three dimensions of professional identity, this research posits the following hypotheses:

**Hypothesis** **H1a.** *Professional cognition positively influences academic self-efficacy.*

**Hypothesis** **H1b.** *Professional emotion positively influences academic self-efficacy.*

**Hypothesis** **H1c.** *Professional appraisal positively influences academic self-efficacy.*

### 2.3. Learning Engagement

The term “engagement” initially found application in the service industry [37] before gradually expanding into various domains. When considering students as the focus of research, the concept of learning engagement emerged. Specifically, it denotes the positive and comprehensive mental state of students during the learning process, assessed through the three dimensions of vitality, dedication, and concentration [38]. This definition has undergone refinement and evolution in research. According to Kuh et al., learning engagement represents the time, energy, and effort students invest in attaining educational goals, underscoring the significance of learning objectives [39]. Connell and Wellborn define learning engagement as the manifestation of intensity and emotional quality in students’ initiation and execution of learning activities [40]. Subsequently, Fredricks et al. introduced three distinct dimensions of behavior, emotion, and cognition [41]. As Internet technology has advanced, an increasing number of studies have focused on evaluating students’ psychological and behavioral engagement [42], asserting the pivotal role of psychological engagement alongside behavioral engagement [43]. This paper places greater emphasis on students’ spiritual learning engagement, accentuating their subjective initiative, with vitality (perseverance in the learning process), dedication (a profound enthusiasm for learning and self-honor), and concentration (focused attention on learning and deriving pleasure from it) as measurement dimensions.

An individual’s attitude toward a specific profession is influenced by their cognitive state and psychological processes [44,45]. The intensity of identity impacts students’ familiarity, affinity, and confidence in the target field [46]. According to the Control-Value Theory (CVT), cognition shapes emotional changes [47], and students develop professional cognition through the study of professional theories and related knowledge. Cognition propels emotional changes and establishes emotional connections, thereby influencing learning motivation and autonomy [11]. This process aligns with the “cognitive-emotion-behavior” theory, illustrating that cognition shapes emotional changes and, in turn, influences students’ learning engagement. Students possessing a high degree of professional identity can effectively sustain elevated levels of learning enthusiasm, thereby maintaining learning engagement [3,48,49]. Conversely, a low degree of professional recognition tends to result in a moderate or lower level of college students’ learning engagement [50]. The research scope extends from students in a specific program to encompass all university students [51,52]. The aforementioned studies indicate that the higher students’ professional identity, the more positive emotional value they generate, thus enhancing learning engagement, and vice versa. Building upon this, the following hypotheses are proposed:

**Hypothesis** **H2a.** *Professional cognition positively influences learning engagement*.

**Hypothesis** **H2b.** *Professional emotion positively influences learning engagement.*

**Hypothesis** **H2c.** *Professional appraisal positively influences learning engagement.*

Academic self-efficacy proves to be a more reliable predictor of academic achievement than other cognitive and affective processes [53], exerting a significant influence on learning and behavioral performance [54]. According to Bandura’s self-efficacy theory, students form anticipated judgments about their abilities in the learning process, consequently influencing their learning engagement [55]. Students endowed with high academic self-efficacy exhibit increased confidence in task completion, leading to more active participation and, thereby, enhancing learning engagement [56,57,58]. Learning engagement primarily manifests as an external behavioral display, while academic self-efficacy manifests as an internal sense of accomplishment [59]. Academic self-efficacy stands as the core driving force behind students’ development [60], positively promoting their learning engagement [61]. Lin [62] demonstrated the predictive power of multifaceted self-efficacy on various aspects of learning engagement. In e-learning, academic self-efficacy directly and indirectly influences learning engagement [63]. Students with high self-efficacy possess a relatively accurate judgment of their ability to complete learning tasks, thereby determining the level of commitment they should dedicate to task completion. Positive emotional motivation propels students to achieve higher levels of learning engagement, resulting in favorable learning outcomes. Consequently, the following hypothesis is proposed:

**Hypothesis** **H3.** *Academic self-efficacy positively influences learning engagement.*

### 2.4. University Support

The university environment is crucial for students’ academic and social-emotional development [13], playing a vital role in their adaptation [64]. University support encompasses learning assistance, social support, academic resources, and fundamental facilities provided by colleges and universities to meet the comprehensive development needs of students, contributing to the realization of university development goals [65]. For instance, Azila-Gbettor examined the relationship between university support and students’ psychological, academic, and social adjustment during the COVID-19 period [15]. University support constitutes a significant component of social support available to students [66], playing a pivotal role in their overall development. Day asserted that university support can mitigate prejudice-based bullying [16], and Guo investigated its impact on reducing depression among bullied students in the Chinese context [17]. The aforementioned research delved into the influence of university support on students’ depression [67]. Simultaneously, students’ perceived university support is linked to heightened learning motivation, lower attrition rates, and a sense of belonging to the university, resulting in lower levels of depressive symptoms [68,69] and fostering a qualitative improvement in the learning experience [70]. Hence, it is imperative to acknowledge the profound impact of university support on students’ learning outcomes.

University support significantly influences students’ academic self-efficacy. Learning within an uncertain environment may diminish academic self-efficacy [71], and a high level of university support has been shown to enhance students’ academic self-efficacy [72]. Notably, there exists a positive correlation between student negotiation and self-efficacy [73]. Peer support indirectly impacts the depth of online learning, with its effects mediated by emotion and self-efficacy [74]. Positive relationships with both teachers and classmates play a crucial role in alleviating students’ negative emotions, reducing adverse physical and mental pressures, and consequently fostering higher academic self-efficacy [75]. Supported by teachers and peers, students endeavor to bridge the gap between reality and learning goals [76], forming a correct judgment of their own abilities and, in turn, elevating their academic self-efficacy [77]. Thus, this study proposes the following hypothesis:

**Hypothesis** **H4.** *University support positively influences academic self-efficacy.*

Social background plays a pivotal role in personal growth and development. When the social background can support the satisfaction of individual needs, individuals are more likely to invest time and energy in achieving goals [78], leading to higher engagement in learning [79]. Conversely, the opposite of higher learning engagement is learning burnout. Current research has demonstrated that in the absence of university support and interaction, students are more likely to experience burnout in professional learning activities [80]. Administrative support from colleges and universities can effectively alleviate students’ learning burnout [81]. Factors such as the university’s learning atmosphere, scale, and sense of belonging impact students’ learning engagement [82,83]. Support from teachers and peers in the university environment is also crucial for students [13,41]. Teacher support can effectively promote students’ emotional and cognitive engagement [84]. When students feel supported by teachers, they experience stronger satisfaction and higher learning interest, leading to increased learning engagement. Peer support exhibits a strong correlation with students’ learning engagement [85]. When students enter a peer group with a higher level of engagement, their own engagement level significantly improves [86]. The mutual pressure among peers motivates students to engage more deeply in learning to integrate with their peers. Therefore, university support has a significant correlation with students’ learning engagement. Consequently, the hypothesis is proposed:

**Hypothesis** **H5.** *University support positively influences learning engagement*.

**Figure 1:**
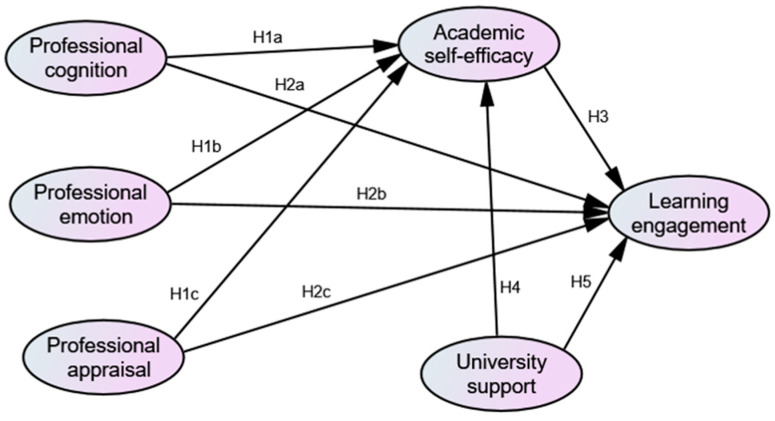
Theoretical Model.

Figure 1 presents the theoretical model of the hypothesized relationships among professional identity, academic self-efficacy, learning engagement, and perceived university support. 

## 3. Materials and Methods

### 3.1. Survey Instrument

Based on the literature review, a questionnaire was developed to measure professional identity, self-efficacy, learning engagement, and perceived university support. The questionnaire consists of five parts: (1) professional identity, (2) academic self-efficacy, (3) learning engagement, (4) university support, and (5) socio-demographic information. In particular, professional identity was measured using a scale adapted from previous work [3,87]. It includes three subscales: cognition, emotion, and appraisal. Self-efficacy was assessed with ten items adapted from the validated Chinese version of the General Self-Efficacy Scale developed by Schwarzer et al. [88]. Learning engagement was assessed using the Utrecht Work Engagement Scale (UWES) [89]. It contains three subscales (i.e., vigor, dedication, and absorption), and each subscale is evaluated with 3 items. For analysis, 9 items from the three subscales were treated as a single predictor of learning engagement following previous work’s recommendation [37,90]. The UWES-S scale was approved with high reliability and validity in previous studies for Chinese students [3,90]. The measurement of university support was modified from the scale developed by Saeed et al. [91], which contains 13 items. The specific measurement items were shown in Appendix A. All the above measurement items were scored on a 5-point Likert scale from 1 (strongly disagree) to 5 (strongly agree).

### 3.2. Data Collection

The sample for this study was tourism management students currently enrolled in a 4-year bachelor’s degree program in China. A snowball sampling method was utilized in this study. The research team first approached tourism students from a large public university in Jiangsu Province, China. These tourism students were then asked to assist in recruiting respondents and were encouraged to share the online survey link with tourism students studying at other universities in China. According to Oh and Pyo [92], the snowball sampling method is useful in increasing generalizability by sampling participants across diverse backgrounds. The data collection took approximately three months to complete, from March to May 2023. A total of 333 valid questionnaires were obtained and used for data analysis after the screening. 

### 3.3. Data Analysis

SPSS 23.0 and SmartPLS 4.0 were employed in this study to analyze the data. Specifically, SPSS 23.0 was first adopted to analyze the demographic profiles of respondents as well as to examine the quality of the data. According to Gotz et al. [93], PLS-SEM is a well-established technique effective for both predictive applications and theory building. Therefore, after the assessment of missing data, outliers, and normality through SPSS 23.0, SmartPLS 4.0 was used to evaluate the reliability and validity of measurement and structural models and to assess the hypothesized relationships among the constructs.

## 4. Results

### 4.1. Profile of Respondents

The descriptive analysis showed that among the 333 respondents in this study, 75.80% were females and 24.20% were males. This finding is in line with previous studies indicating there were more female students than male students enrolled in the tourism program in China [3,94]. As for the grade distribution, freshman and sophomore respondents accounted for 21.10% and 30.00%, respectively, and junior and senior respondents accounted for 26.70% and 22.20%, respectively. 

### 4.2. Common Method Bias

In order to confirm common method variance is not a concern in the current study, a post hoc Harman’s single-factor analysis was adopted following Podsakoff et al.’s [95] recommendation. Specifically, the analysis was conducted to check that the amount of variance in observed variables was not explained by one single factor. The results showed the variance accounted for 35.23%, below the suggested threshold of 40.00%. Therefore, common method bias is not a concern in this study.

### 4.3. Assessment of Measurement Model

To evaluate the reliability and validity of the constructs adopted in this study, internal consistency reliability, indicator reliability, convergent validity, and discriminant validity were assessed. Specifically, in terms of measurement model reliability, Cronbach’s alpha (α), factor loading, and composite reliability (CR) were assessed. According to Table 1, the Cronbach’s alpha values of constructs ranged from 0.900 to 0.962, and the composite reliability values ranged from 0.902 to 0.962, both exceeding the thresholds of 0.700. In addition, all factor loadings are above the minimum level of 0.700. Thus, the data showed satisfactory internal consistency reliability and indicator reliability. As for the model validity, the value of the average variance extracted (AVE) of all constructs is above 0.50, suggesting satisfactory convergent validity (Table 1). Moreover, the square root of the AVE values exceeded the correlation between variables, confirming the discriminant validity of the model (as shown in Table 2). Therefore, the measurement model proved to have sufficient reliability and validity. 

### 4.4. Assessment of the Structural Model

First, it is significant to confirm there are no collinearity issues between the constructs. The variance inflation factor (VIF) values for each construct are less than the threshold of 5, indicating that multicollinearity is not a concern in the structural model. Second, the coefficient of determination (R^2^ value) and Stone-Geisser’s Q^2^ value were calculated to evaluate the predictive validity and relevance of the structural model. According to Table 3, with R^2^ values of the constructs all above the suggested threshold of 0.10 [96] and Q^2^ values of the constructs higher than the threshold of 0.00 [97], the structural model was perceived to have sufficient predictive validity and relevance. Third, the estimated path coefficients and t-statistics of the hypotheses were examined using a bootstrapping approach. Table 4 presents the results of the hypothesized model relationships.

**Figure 2:**
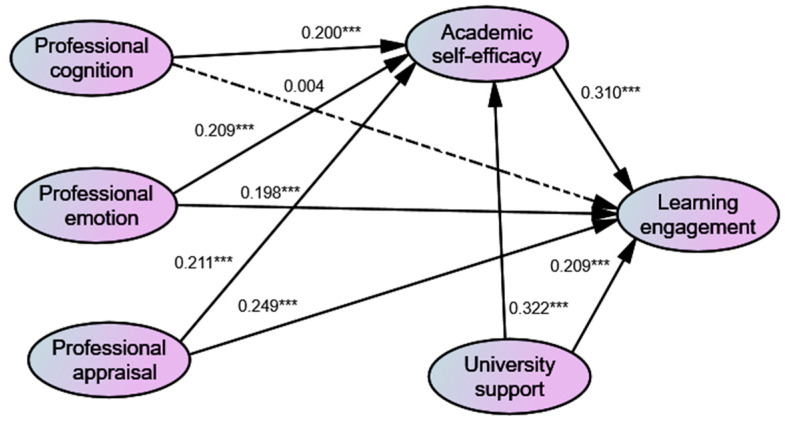
Results of the structural model. Note: *** Significant at *p* < 0.01.

Specifically, Hypotheses 1a, 1b, and 1c predicted the positive influences of students’ professional cognition, professional emotion, and professional behavior on learning engagement. The results revealed that Hypothesis 1a was accepted at β = 0.200, *p* < 0.01, Hypothesis 1b was accepted at β = 0.209, *p* < 0.01, and Hypothesis 1c was accepted at β = 0.211, *p* < 0.01. The relationship between students’ professional cognition and learning engagement was found to be not significant (β = 0.004, *p* > 0.05), rejecting Hypothesis 2a. However, students’ professional emotion and professional appraisal were confirmed to have positive impacts on learning engagement, supporting Hypothesis 2b at β = 0.198, *p* < 0.01, and Hypothesis 2c at β = 0.249, *p* < 0.01, respectively. Next, students’ academic self-efficacy was confirmed to be positively related to learning engagement (β = 0.310, *p* < 0.01), supporting Hypothesis 3. Hypothesis 4 and Hypothesis 5 assumed the positive influences of students’ perceived university support on their academic self-efficacy and learning engagement, respectively. The results showed that the direct impacts of university support on self-efficacy (β = 0.322, *p* < 0.01) and learning engagement (β = 0.209, *p* < 0.01) were significantly positive. Therefore, Hypothesis 4 and 5 were accepted. Figure 2 below presents the results of the structural model. 

### 4.5. Indirect and Mediating Effects

In addition to the examination of the direct relationships among variables, indirect effects were also tested. In particular, multiple mediation analyses were conducted to examine the role of academic self-efficacy in mediating the influence of professional identity and university support on learning engagement. To test the mediating effect of academic self-efficacy, the bootstrapping procedure with 5000 samples was employed. Table 4 presents the results of indirect and mediating effects. 

According to Table 5, results showed that professional cognition and professional emotion significantly and indirectly influence learning engagement (β = 0.058, *p* < 0.01; β = 0.079, *p* < 0.05) through academic self-efficacy, supporting the significant mediating role of academic self-efficacy. In particular, according to Hair [98], the variance accounted for (VAF) value between 20% and 80% indicates partial mediation, and over 80% indicates full mediation. Therefore, as shown in Table 5, academic self-efficacy plays a full mediating role between professional cognition and learning engagement, as well as a partial mediating role between professional emotion and learning engagement. However, the indirect effect of professional appraisal no learning engagement through academic self-efficacy was not significant (β = 0.054, *p* > 0.05). This finding suggested that academic self-efficacy was not a significant mediator between professional appraisal and learning engagement. Lastly, the results verified a significant partial mediating role of academic self-efficacy between university support and learning engagement (β = 0.096, *p* < 0.01).

## 5. Discussion and Conclusions

This study aims to investigate the relationships among tourism students’ professional identity, academic self-efficacy, learning engagement, and university support. The following conclusions are drawn: (1) The relationship between students’ professional cognition and learning engagement was not significant, while students’ professional emotion and professional appraisal positively influenced learning engagement. (2) All three dimensions of professional identity positively influenced students’ academic self-efficacy. (3) Students’ academic self-efficacy had a positive effect on learning engagement, and students’ perceived university support positively influenced both learning engagement and academic self-efficacy. The present findings have both theoretical and practical implications, which are listed below.

### 5.1. Theoretical Implications

Firstly, this study enhances the understanding of the relationship between professional identity and learning engagement in tourism students. While numerous studies focus on the connection between specific individual or environmental variables and learning engagement—such as academic self-efficacy; professional identity; and university support—these studies often singularly examine the impact of a solitary variable on students’ learning engagement; with scant attention given to tourism students. In particular, Chen et al. [8] believed that professional identity can significantly influence academic self-efficacy, and Lin [62] proved that self-efficacy has a positive impact on learning engagement. Zhang et al. [59] suggested that academic self-efficacy would drive an internal sense of achievement, thus promoting students’ learning engagement [61]. Professional identity is often positively correlated with learning engagement [3,50]. In the study on professional engagement, academic self-efficacy, and learning engagement of nursing students, Zhou [36] verified the positive impact of professional identity on academic self-efficacy and learning engagement and again emphasized the mediating role of academic self-efficacy. In addition, in the study of Turner [77], through the diachronic exploration of teacher support in university support, it was concluded that teacher support can improve students’ academic self-efficacy. Jacobs et al.‘s study also proves that a high level of college support can positively promote academic self-efficacy [72]. University support provides students with the necessary conditions to continue learning and also influences students’ learning engagement to a certain extent [79]. Recognizing that learning engagement is likely influenced by a blend of personal, professional, and university factors, this study addresses this gap by systematically exploring the interplay among multiple variables for tourism students. Notably, the study validates a theoretical model that investigates the relationships among undergraduate tourism students’ professional identity, academic self-efficacy, learning engagement, and university support.

Secondly, this research broadens the scope of inquiries into students’ professional identities. Wang [51] et al. explored the relationship between professional identity and the learning engagement of medical students. Chen and Yang [8] explored the relationship between professional identity, learning burnout, and academic self-efficacy for special education students. While existing research on students’ professional identity predominantly centers on students in medical and educational programs, little consideration is given to tourism students. DeSimone et al.’s research shows that professional identity can influence students’ learning confidence in the field [45], and high professional identity can promote students’ learning enthusiasm and thus enhance their learning engagement [3]. Undergraduate students in tourism programs exhibit a high rate of program changes, coupled with a notable deficiency in their professional identity. This lack of professional identity detrimentally impacts their learning engagement, potentially diminishing their competitiveness in the talent market and impeding the development of China’s tourism industry. By shedding light on the professional identity of tourism students, this study makes valuable contributions toward improving their learning engagement.

Thirdly, this study validates the specific dimensions of professional identity. Building on the work of Yu [3], who identified three dimensions—professional cognition; professional appraisal; and professional emotion—this research further substantiates these dimensions for students in tourism programs. Additionally, by delving into the nuances of these dimensions, the study advances our comprehension of the relationships among professional identity, academic self-efficacy, learning engagement, and perceived university support.

### 5.2. Practical Implications

Firstly, this study’s findings yield practical implications for the field of tourism education. Specifically, they offer valuable insights for university administrators seeking to comprehend tourism students’ perspectives on their programs, as well as their levels of learning engagement and academic self-efficacy. Notably, students’ professional cognition demonstrates no direct correlation with learning engagement, whereas students’ professional emotion and professional appraisal exert positive influences on learning engagement. Consequently, universities should not only prioritize the enhancement of students’ professional emotion and professional appraisal to elevate their learning engagement but also consider adjustments to the professional curriculum. Implementing targeted measures to foster learning engagement is essential. Concurrently, universities can encourage instructors to reflect on the teaching process and adopt diverse teaching methods to bolster students’ enthusiasm and confidence, motivating them to actively engage in their studies.

Secondly, this study’s findings contribute to fostering robust interactive relationships between universities and students. Recognizing that universities play a pivotal role in nurturing students’ academic prowess and emotional well-being, these aspects are integral to the exploration of students’ learning engagement. University support is an important part of the social support available to students [66]. Azila-Gbettor has verified that university support can influence students’ psychological state and academic performance during COVID-19 [15]. Morley [68] indicated that the greater university support perceived by students, the lower the level of depression and thus the higher the learning engagement. Universities should strive to provide tangible and perceptible support services, thereby fostering students’ learning engagement and academic self-efficacy. In reciprocity, students’ enhanced learning engagement becomes a catalyst for the ongoing development of the university.

Thirdly, enhancing the learning engagement of tourism students proves beneficial for the growth of the tourism industry. Wu et al. stated that the intervention of students’ intrinsic learning motivation can promote students to better improve their learning effectiveness [99] and have higher professional skills. This study examines the multifaceted factors influencing students’ learning engagement, contributing to the effective cultivation of high-quality tourism talents. The results show that professional cognition has no significant impact on learning engagement, while professional emotion and professional appraisal have a positive impact on learning engagement. Academic self-efficacy has a positive impact on learning engagement, and university support has a positive impact on learning engagement and academic self-efficacy. Exploring the above factors can effectively improve students’ learning effectiveness. In addition, by doing so, it enables a more substantial number of skilled professionals to contribute to and serve the thriving tourism industry.

### 5.3. Limitations and Further Research Suggestions

This study also suffers from some limitations. First, this research focuses on tourism students in China; however, whether the findings apply to students in other programs and other countries remains unexplored. Future research could further test the model across students in different programs and different countries. Second, this study investigated the interrelationships among professional identity, academic self-efficacy, learning engagement, and perceived university support using a quantitative method. The interpretation of the results is somewhat restricted. Future research could utilize a mixture of both quantitative and qualitative research methods to generate more insights into the relationships. Third, given the cross-sectional nature of this study, the analysis of causal relationships in this study also bears limitations. It would be meaningful for future research to conduct a longitudinal study to examine the causal relationships of the research model.

## Figures and Tables

**Table 1:** Assessment of the measurement model.

Constructs	Items	Loading	Cronach’s Alpha	Composite Reliability	Average Variance Extracted
Professional Cognition	PC1	0.860	0.900	0.902	0.714
	PC2	0.848			
	PC3	0.837			
	PC4	0.808			
	PC5	0.869			
Professional Emotion	PE1	0.868	0.924	0.927	0.654
	PE2	0.839			
	PE3	0.814			
	PE4	0.819			
	PE5	0.767			
	PE6	0.799			
	PE7	0.704			
	PE8	0.849			
Professional Appraisal	PA1	0.811	0.921	0.922	0.718
	PA2	0.856			
	PA3	0.837			
	PA4	0.844			
	PA5	0.870			
	PA6	0.867			
Learning engagement	LE1	0.735	0.910	0.914	0.582
	LE2	0.777			
	LE3	0.749			
	LE4	0.801			
	LE5	0.836			
	LE6	0.775			
	LE7	0.713			
	LE8	0.711			
	LE9	0.762			
Academic Self-efficacy	SE1	0.793	0.942	0.943	0.656
	SE2	0.774			
	SE3	0.786			
	SE4	0.821			
	SE5	0.832			
	SE6	0.833			
	SE7	0.756			
	SE8	0.872			
	SE9	0.851			
University support	SE10	0.778			
	US1	0.826	0.962	0.962	0.686
	US2	0.826			
	US3	0.826			
	US4	0.810			
	US5	0.830			
	US6	0.812			
	US7	0.816			
	US8	0.820			
	US9	0.850			
	US10	0.857			
	US11	0.822			
	US12	0.821			
	US13	0.851			

**Table 2:** Discriminant validity.

	PA	PC	PE	LE	SE	US
PA	0.848					
PC	0.783	0.845				
PE	0.871	0.852	0.809			
LE	0.809	0.760	0.824	0.763		
SE	0.774	0.777	0.807	0.823	0.810	
US	0.719	0.735	0.746	0.774	0.772	0.828

Note: The square root of AVE is shown on the diagonal of the matrix.

**Table 3:** Coefficients of determination (R^2^) and prediction variance (Q^2^) of the construct.

Endogenous Latent Construct	Coefficients of Determination (R^2^)	Predictive Relevance (Q^2^)
Learning engagement	0.781	0.445
Academic self-efficacy	0.733	0.476

**Table 4:** Results of structural model and hypotheses testing.

Hypothesized Relationships	Coefficient	Results
H1a: Professional Cognition → Academic Self-efficacy	0.200 ***	Supported
H1b: Professional Emotion → Academic Self-efficacy	0.209 ***	Supported
H1c: Professional Appraisal → Academic Self-efficacy	0.211 ***	Supported
H2a: Professional Cognition → Learning engagement	0.004	Not supported
H2b: Professional Emotion → Learning engagement	0.198 ***	Supported
H2c: Professional Appraisal → Learning engagement	0.249 ***	Supported
H3: Academic Self-efficacy → Learning engagement	0.310 ***	Supported
H4: University support →Academic Self-efficacy	0.322 ***	Supported
H5: University support → Learning engagement	0.209 ***	Supported

Note: *** Significant at *p* < 0.01.

**Table 5:** Indirect and mediating effects.

	Original Sample (O)	Standard Deviation (STDEV)	T Statistics	*p* Values	Variance Accounted for Value (VAF)	Result
Professional cognition → Academic self-efficacy → Learning engagement	0.058	0.022	2.661	0.008	93.548%	Full mediation
Professional emotion → Academic self-efficacy → Learning engagement	0.079	0.033	2.402	0.016	26.246%	Partial mediation
Professional appraisal → Academic self-efficacy → Learning engagement	0.054	0.028	1.950	0.051	19.148%	No mediation
University support → Academic self-efficacy → Learning engagement	0.096	0.029	3.323	0.001	31.893%	Partial mediation

## Data Availability

Data are unavailable due to privacy restrictions.

## Appendix A

**Table A1:** Questionnaire. All items are designed and measured on a 5-point Likert scale of agreement (where 1 denotes strongly disagree and 5 denotes strongly agree).

Construct	Measurement Items
Professional cognition	I know what the tourism program requires of students.
	I understand the employment situation in the tourism program.
	I know what the outside world says about the tourism program.
	I know the position of tourism program in our university.
	In general, I have a clear understanding of the tourism program.
Professional emotion	I am willing to engage in work related to the tourism program.
	I have accepted the tourism program in my heart.
	I haven’t thought about changing my program.
	I have a positive evaluation of the tourism program.
	I have great confidence in the development prospects of the tourism program.
	I have developed positive feelings towards the tourism program.
	I am satisfied with the overall situation of the tourism program at our university.
	In general, I like tourism programs.
Professional appraisal	I have good professional thinking in tourism.
	My personality matches my program in tourism.
	The tourism program can reflect my expertise.
	I am persistent in my study of tourism.
	I am well suited to study tourism.
	I feel at ease studying tourism.
Academic self-efficacy	I can always manage to solve difficult problems in my studies if I try hard enough.
	If someone opposes me, I can find means and ways to get what I want in studying.
	It is easy for me to stick to my study aims and accomplish my academic goals.
	I am confident that I could deal efficiently with unexpected events during my studies.
	Thanks to my resourcefulness, I know how to handle unforeseen situations in study.
	I can solve most study problems if I invest the necessary effort.
	I can remain calm when facing difficulties in studying because I can rely on my coping abilities.
	When I am confronted with a problem in my study, I can usually find several solutions.
	If I am in a bind, I usually have something to do.
	No matter what comes my way, I’m usually able to handle it.
Learning engagement	I am very resilient mentally, as far as my studies are concerned.
	I feel strong and vigorous when I’m studying or going to class.
	When I’m doing my work as a student, I feel bursting with energy.
	I am enthusiastic about my studies.
	I am proud of my studies.
	I find my studies full of meaning and purpose.
	When I am studying, I forget everything else around me.
	I am immersed in my studies.
	It is difficult to detach myself from my studies.
University support	My university offers professional courses on tourism.
	My university offers project work focused on tourism.
	My university offers internship focused on tourism.
	My university offers a bachelor’s or master’s degree in tourism.
	My university arranges conferences/workshops on tourism.
	My university brings tourism students into contact with each other.
	My university creates awareness of tourism as a possible career choice.
	I feel very comfortable with the overall teaching and learning environment, atmosphere, equipment, and facilities of my university.
	The library of my university has abundant and authoritative information and an excellent environment, which can meet my learning needs.
	I can always get notice and information related to my tourism program in a timely and convenient way.
	The teachers/mentors at my university will take the initiative to praise and affirm my progress in my tourism studies.
	The teachers/mentors at my university will actively listen to my confusion in tourism studies and give me comfort.
	The faculty or teaching support staff of my university will give me advice and guidance for further study.
